# A generalisation of the method of regression calibration

**DOI:** 10.1038/s41598-023-42283-y

**Published:** 2023-09-13

**Authors:** Mark P. Little, Nobuyuki Hamada, Lydia B. Zablotska

**Affiliations:** 1 Radiation Epidemiology Branch, National Cancer Institute, Room 7E546, 9609 Medical Center Drive, Bethesda, MD 20892-9778 USA; 2 Biology and Environmental Chemistry Division, Sustainable System Research Laboratory, Central Research Institute of Electric Power Industry (CRIEPI), 1646 Abiko, Chiba, 270-1194 Japan; 3 Department of Epidemiology and Biostatistics, School of Medicine, University of California, San Francisco, 550 16th Street, 2nd Floor, San Francisco, CA 94143 USA

**Keywords:** Mathematics and computing, Software, Statistics, Epidemiology, Natural hazards

## Abstract

There is direct evidence of risks at moderate and high levels of radiation dose for highly radiogenic cancers such as leukaemia and thyroid cancer. For many cancer sites, however, it is necessary to assess risks via extrapolation from groups exposed at moderate and high levels of dose, about which there are substantial uncertainties. Crucial to the resolution of this area of uncertainty is the modelling of the dose–response relationship and the importance of both systematic and random dosimetric errors for analyses in the various exposed groups. It is well recognised that measurement error can alter substantially the shape of this relationship and hence the derived population risk estimates. Particular attention has been devoted to the issue of shared errors, common in many datasets, and particularly important in occupational settings. We propose a modification of the regression calibration method which is particularly suited to studies in which there is a substantial amount of shared error, and in which there may also be curvature in the true dose response. This method can be used in settings where there is a mixture of Berkson and classical error. In fits to synthetic datasets in which there is substantial upward curvature in the true dose response, and varying (and sometimes substantial) amounts of classical and Berkson error, we show that the coverage probabilities of all methods for the linear coefficient $\alpha$ are near the desired level, irrespective of the magnitudes of assumed Berkson and classical error, whether shared or unshared. However, the coverage probabilities for the quadratic coefficient $\beta$ are generally too low for the unadjusted and regression calibration methods, particularly for larger magnitudes of the Berkson error, whether this is shared or unshared. In contrast Monte Carlo maximum likelihood yields coverage probabilities for $\beta$ that are uniformly too high. The extended regression calibration method yields coverage probabilities that are too low when shared and unshared Berkson errors are both large, although otherwise it performs well, and coverage is generally better than these other three methods. A notable feature is that for all methods apart from extended regression calibration the estimates of the quadratic coefficient $\beta$ are substantially upwardly biased.

## Introduction

Cancer risks following exposure to moderate and high levels of radiation dose are reasonably well understood^1,2^. There are beginning to be studies yielding direct estimates of radiation risk at low dose (< 100 mGy) low-linear energy transfer (LET) radiation^3–6^. This is particularly the case for highly radiogenic sites such as thyroid^3^ and leukaemia^4^. For most other cancer endpoints it is necessary to assess risks via extrapolation from groups exposed at moderate and high levels of dose. A number of recent reviews of low dose risk have been conducted, in particular those by the National Council on Radiation Protection and Measurements (NCRP)^7^ and by the National Cancer Institute^8–13^. A major source of uncertainty in assessment of low dose risk concerns the extrapolation of risks at high doses and high dose-rates to those at low doses (< 0.1 gray (Gy)) and low dose-rates (< 5 mGy/hour)^14^. Crucial to the resolution of this area of uncertainty is the modelling of the dose–response relationship and the importance of both systematic and random dosimetric errors for analyses of the dose response, in particular in the Japanese atomic bomb survivors, which is central to evaluations of population risks by a number of committees assessing radiation risk^1,15^. The problem of allowing for measurement error in dose when estimating dose–response relationships has been the subject of much interest in epidemiology^16–31^. A recent review paper summarises at least some of the methods that have been used^32^. It is well recognised that measurement error can alter substantially the shape of this relationship and hence the derived population risk estimates^33^. A method that has been frequently used to correct for the effects of classical error is regression calibration, in which the terms for true dose, $D_{i}$, in regression models are replaced by the condititonal expectation of true dose given the oberved dose $d_{i}$, $E[D_{i} |d_{i} ]$^33^. Regression calibration works well when the magnitude of errors is modest, and when the dose response is not substantially non-linear^33^. When errors are larger methods that take account of the full error distribution such as Monte Carlo maximum likelihood (MCML)^25–27,31^ or Bayesian Markov Chain Monte Carlo (MCMC)^22–24,30^ are likely to perform better.

Dose measurement errors can arise in a number of different ways. In radiotherapy (RT), for example, a machine may be used for delivering radiation doses, $D_{i}$, to a patient, and these true values are randomly distributed around the measured dial setting on the RT machine, $d_{i}$, with error $U_{i}$, so that $D_{i} = d_{i} + U_{i}$, implying that the $d_{i} ,U_{i}$ are independent, i.e., the Berkson error model. Alternatively, the measured “doses”, $d_{i}$ can be distributed at random around the true “doses”, $D_{i}$, so that $d_{i} = D_{i} + U_{i}$ so that the $D_{i} ,U_{i}$ are independent, i.e., the “classical” error model. Although these models look very similar, they are different. In particular the crucial difference is that in the Berkson model the nominal dose and error are independent, but in the classical error model it is the true dose and the error that are independent. In the atomic bomb survivors, radiation doses are estimated by using estimates of the position of the survivors in each city, orientation with respect to the bomb and other shielding structures, e.g., buildings. In this case the estimated doses, $d_{i}$, are thought to be lognormally distributed around the true doses, $D_{i}$ (i.e. classical error model)^34^. This assumption underlies many of the attempts that have been made to model dose error in the Japanese atomic bomb survivor Life Span Study (LSS) data^16–20,22–24,30^. However, some components of assessed dose to the atomic bomb survivors may be associated with Berkson error, for example that associated with estimation of the atomic bomb source term. Some attempts have been made to model this statistically^35^. Methods have been devised that allow for a combination of Berkson and classical errors in the LSS data^36,37^; although shared errors have not been explicitly modelled in the LSS they undoubtedly exist, as for example in the estimates of the bomb yield in the two cities. It is known that regression calibration can work well in cases when dose errors are not substantial and in which there is no curvature in the dose response^33^. However, it is also appreciated that there can be substantial bias in regression calibration when dose errors are substantial, also when errors are non-differential^33,38,39^.

We propose a modification of the regression calibration method which is particularly suited to studies in which there is a substantial amount of shared error, and in which there may also be curvature in the true dose response. We compare the performance of this and other methods for dose error correction using synthetic data closely modelled on the Japanese atomic bomb survivor data^40^.

## Methods

### Synthetic data used for assessing corrections for dose error

We used the publicly available version of the leukaemia and lymphoma data of Hsu et al.^40^ to guide construction of a synthetic dataset, which we provide in outline in Table 1. Specifically we used the person year distribution by bone marrow dose groups 0–0.07, 0.08–0.19, 0.20–0.99, 1.00–2.49, ≥ 2.50 Gy. The central estimates of dose we assumed are close to the person year weighted means of these groups, and as given in Table 1, although for the uppermost dose group we assigned a central estimate of 2 Gy. The numbers of persons are close to the scaled sum of person years in these dose groups, scaling by a factor of 0.002. We assumed a composite Berkson-classical error model in which the true dose $D_{true,i,j}$ and the surrogate dose $D_{surr,i,j}$ to individual $i$ (in dose group $k_{i}$) in simulation $j$ are given by:

1 $$D_{true,i,j} = D_{{cent,k_{i} }} \exp \left[ { - 0.5(\sigma_{share,Berkson}^{2} + \sigma_{unshare,Berkson}^{2} )} \right]\exp \left[ {\sigma_{share,Berkson} \varepsilon_{j} + \sigma_{unshare,Berkson} \delta_{i,j} } \right]$$

2 $$D_{surr,i,j} = D_{{cent,k_{i} }} \exp \left[ { - 0.5(\sigma_{share,Class}^{2} + \sigma_{unshare,Class}^{2} )} \right]\exp \left[ {\sigma_{share,Class} \mu_{j} + \sigma_{unshare,Class} \kappa_{i,j} } \right]$$

**Table 1:** Assumed distribution of persons by radiation dose group, based in part on distribution of person years in the Japanese atomic bomb survivor Life Span Study^40^.

Dose group	Central estimate of dose (Gy)	Scaled numbers of persons
1	0.01	2591
2	0.1	334
3	0.5	438
4	1.5	102
5	2	6

The variables $\varepsilon_{j} ,\delta_{i,j} ,\mu_{j} ,\kappa_{i,j}$ are independent identically distributed $N\left( {0,1} \right)$ random variables. The factors $D_{{cent,k_{i} }} ,D_{{cent,k_{i} }}$ are the central estimates of dose, as given in Table 1. The factors $\exp \left[ { - 0.5(\sigma_{share,Berkson}^{2} + \sigma_{unshare,Berkson}^{2} )} \right]$ and $\exp \left[ { - 0.5(\sigma_{share,Class}^{2} + \sigma_{unshare,Class}^{2} )} \right]$ ensure that the distributions given by (1) and (2) have theoretical mean that coincides with the central estimates $D_{{cent,k_{i} }}$. This composite Berkson-classical error model is suggested by a similar (but purely additive) model proposed by Reeves et al.^21^, whereas the errors in our model are of multiplicative form; the model of course ensures that the simulated doses are always positive. The model has the feature that when the Berkson error geometric standard deviations (GSDs) are set to 0 ($\sigma_{share,Berkson} = \sigma_{unshare,Berkson} = 0$) the model reduces to one with classical error (a mixture of shared and unshared); likewise when the classical error GSDs are set to 0 ($\sigma_{share,Class} = \sigma_{unshare,Class} = 0$) the model reduces to one with pure Berkson error (a mixture of shared and unshared).

We generated a number of different versions of the dose data, with GSD $\sigma_{share,Berkson}$, $\sigma_{unshare,Berkson}$, $\sigma_{share,Class}$, $\sigma_{unshare,Class}$ taking values of 0.2 (20%) or 0.5 (50%). We also explored 4 scenarios with pure classical error, with the Berkson error terms set to 0. This individual dose data was then used to simulate the distribution of $N = 250$ cancers for each of $m = 1000$ simulated datasets, indexed by $j$, using a model in which the assumed probability of being a case for individual $i$ is given by:

3 $$\lambda_{j} [1 + \alpha D_{true,i,j} + \beta D_{true,i,j}^{2} ]$$

the scaling constant $\lambda_{j}$ being chosen for each simulation to make these sum to 1. We assumed coefficients $\alpha = 0.25/{\text{Gy}},\beta = 2/{\text{Gy}}^{2}$, close to the values derived from fits of a similar model to the 237 leukaemias in the data of Hsu et al.^40^.

A total of $m = 1000$ samples were taken of each type of dose, as given by expressions (1) and (2). A total of *n* = 500 simulations of these dose + cancer ensembles were used to fit models and evaluate fitted model means and coverage probability. Having derived synthetic individual level data, for the purposes of model fitting, for all models except MCML, the data were then collapsed (summing cases, averaging doses) into the 5 dose groups given in Table 1. Poisson linear relative risk generalised linear models^41^ were fitted to this grouped data, with rates given by expression (3), using as offsets the number per group in Table 1. Models were fitted using four separate methods:

1. unadjusted – using only the mean surrogate doses per group given by group means of the samples generated by expression (2), using a single sampled dose per individual for each of $m = 500$ dose + cancer ensembles;
2. regression calibration adjusted – using the mean true doses per group given by group means of the samples generated by expression (1), averaged over the $n = 1000$ dose samples, for each of $m = 500$ dose + cancer ensembles;
3. extended regression calibration adjusted – using the mean true doses per group given by group means of the samples generated by expression (1), averaged over the $n = 1000$ dose samples, for each of $m = 500$ dose + cancer ensembles, and with additional adjustments to the likelihood outlined in Appendix A;
4. MCML, using the full set of mean true doses per group, the mean doses per group for each simulation being given by group means of the samples generated by expression (1), averaged over the $n = 1000$ dose samples.

In all cases confidence intervals were derived using the profile likelihood^41^. The Fortran 95-2003 program used to generate these datasets and perform Poisson model fitting, and the relevant steering files employed to control this program are given in online Appendix B.

## Results

As shown in Table 2, the coverage probabilities of all methods for the linear coefficient $\alpha$ are near the desired 95% level, irrespective of the magnitudes of assumed Berkson and classical error, whether shared or unshared. However, the coverage probabilities for the quadratic coefficient $\beta$ are generally too low for the unadjusted and regression calibration methods, particularly for larger magnitudes of Berkson error (with GSD = 50%), whether this is shared or unshared (Table 2). The extended regression calibration method also yields coverage probabilities that are too low when shared and unshared Berkson errors are both large (with GSD = 50%), although otherwise it performs well, and coverage is uniformly better than these other two methods (Table 2). In contrast MCML yields coverage probabilities for $\beta$ that are uniformly too high (Table 2). The interindividual correlations of true dose are generally moderate to high, ranging from 0.15 to 0.84 (Table 2). The correlations between the group mean true doses are generally very high, in all cases > 0.95, for obvious reasons—as a result of the averaging the unshared errors will become relatively much less important than the shared errors (which are unaffected by averaging), and it is these that drive the correlations.

**Table 2:** Coverage probability of profile likelihood confidence intervals for fits of linear-quadratic model.

Magnitude of error distribution (GSD)				Sample Pearson correlation coefficient between individual true doses	Unadjusted model		Regression calibration adjusted		Extended regression calibration adjusted		Monte Carlo maximum likelihood	
					Coverage %		Coverage %		Coverage %		Coverage %	
Unshared Berkson error (%)	Shared Berkson error (%)	Unshared classical error (%)	Shared classical error (%)		*α*	*β*	*α*	*β*	*α*	*β*	*α*	*β*
0	0	20	20	NA	95.0	80.8	95.2	94.8	95.2	94.8	95.2	94.8
0	0	20	50	NA	94.4	55.4	95.2	94.8	95.2	94.8	95.2	94.8
0	0	50	20	NA	94.4	79.6	95.2	94.8	95.2	94.8	95.2	94.8
0	0	50	50	NA	94.6	55.0	95.2	94.8	95.2	94.8	95.2	94.8
20	20	20	20	0.50	95.0	80.0	95.4	94.0	94.8	98.4	95.4	99.0
20	20	20	50	0.50	94.8	53.6	95.4	94.0	94.8	98.4	95.4	99.0
20	20	50	20	0.50	94.6	78.2	95.4	94.0	94.8	98.4	95.4	99.0
20	20	50	50	0.50	94.6	52.2	95.4	94.0	94.8	98.4	95.4	99.0
20	50	20	20	0.84	93.6	77.4	94.4	85.6	95.4	94.8	95.0	100.0
20	50	20	50	0.84	93.2	48.4	94.4	85.6	95.4	94.8	95.0	100.0
20	50	50	20	0.84	93.8	75.4	94.4	85.6	95.4	94.8	95.0	100.0
20	50	50	50	0.84	93.8	48.2	94.4	85.6	95.4	94.8	95.0	100.0
50	20	20	20	0.15	94.2	76.6	94.0	86.0	94.4	94.8	95.0	99.2
50	20	20	50	0.15	93.4	48.4	94.0	86.0	94.4	94.8	95.0	99.2
50	20	50	20	0.15	94.0	75.6	94.0	86.0	94.4	94.8	95.0	99.2
50	20	50	50	0.15	94.0	49.0	94.0	86.0	94.4	94.8	95.0	99.2
50	50	20	20	0.45	95.4	64.0	95.4	67.8	95.0	80.4	96.4	100.0
50	50	20	50	0.45	95.0	40.0	95.4	67.8	95.0	80.4	96.4	100.0
50	50	50	20	0.45	94.4	64.6	95.4	67.8	95.0	80.4	96.4	100.0
50	50	50	50	0.45	94.2	40.0	95.4	67.8	95.0	80.4	96.4	100.0

Coverage probability evaluated using *m* = 500 dose + cancer simulations.

*GSD* geometric standard deviation.

**Figure 1:**
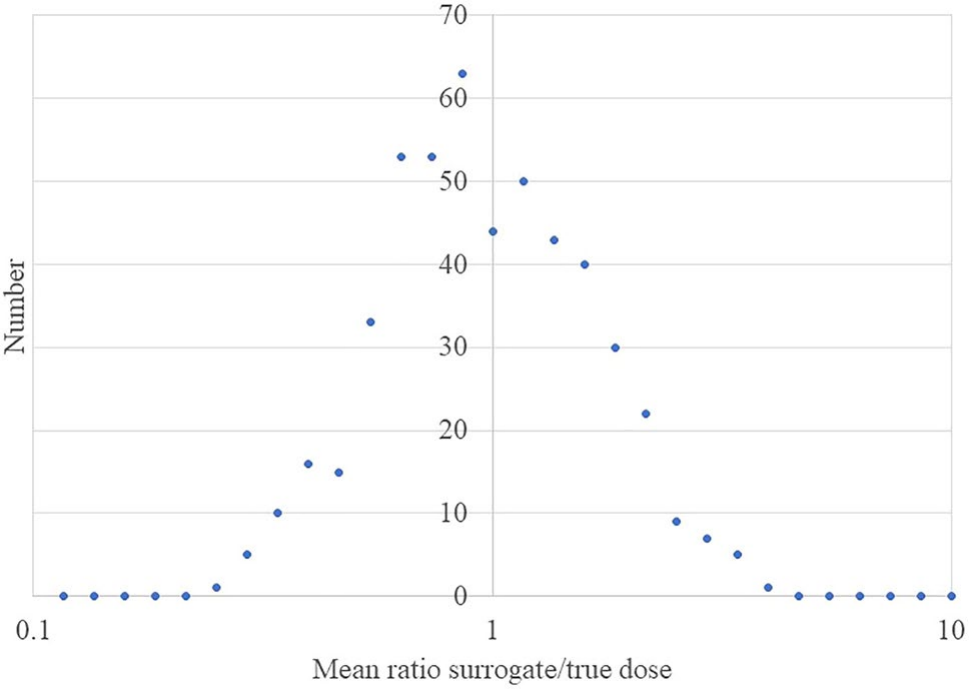
Distribution of weighted mean ratio of surrogate to true dose when there is 50% shared classical error, 50% shared classical error, no Berkson error (as in 4th row of Table 3). A logarithmic X-axis is used, with step size = 10^(1/15)^.

**Figure 2:**
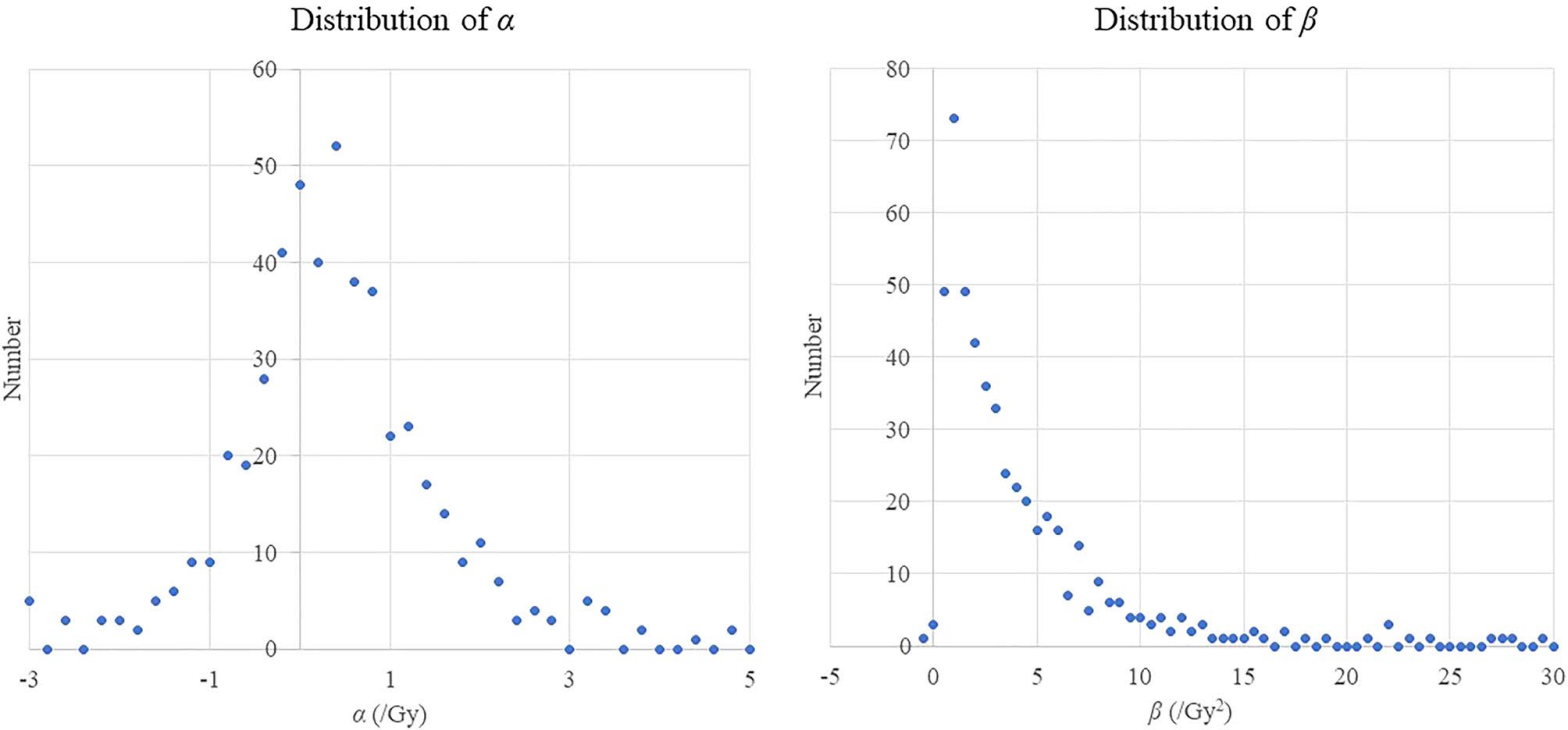
Distribution of fitted linear and quadratic coefficients when there is 50% shared classical error, 50% shared classical error, no Berkson error (as in 4th row of Table 3). The step size used for $\alpha$ is 0.2, the step size used for $\beta$ is 0.5.

Table 3 shows the coefficient mean values, averaged over all 500 simulations. A notable feature is that for all methods apart from extended regression calibration the estimates of the quadratic coefficient $\beta$ are upwardly biased. There is upward bias in estimates of both $\alpha$ and $\beta$ in the unadjusted analysis (using surrogate dose) even when there are no Berkson errors, for various magnitudes of classical errors, as shown by the first four rows of Table 3. As can be seen from Fig. 1, in this case (with shared and unshared classical errors having GSD = 50%) the mean ratio of surrogate to true dose is lognormal in the way one would expect, but as shown in Fig. 2 the fitted $\hat{\alpha }$ and $\hat{\beta }$ are markedly skew, with pronounced upper tail, particularly for $\hat{\beta }$. It is this long upper tail that accounts for the upward bias in both $\hat{\alpha }$ and $\hat{\beta }$ in the unadjusted analysis (using surrogate dose).

**Table 3:** Mean over *m* = 500 dose + cancer simulations of regression coefficients in fits of linear-quadratic model.

Magnitude of error distribution (GSD)				Unadjusted		Regression calibration		Extended regression calibration		Monte Carlo maximum likelihood	
				ERR/Gy	ERR/Gy^2^	ERR/Gy	ERR/Gy^2^	ERR/Gy	ERR/Gy^2^	ERR/Gy	ERR/Gy^2^
Unshared Berkson error (%)	Shared Berkson error (%)	Unshared classical error (%)	Shared classical error (%)	*α*	*β*	*α*	*β*	*α*	*β*	*α*	*β*
0	0	20	20	0.221	2.278	0.196	2.061	0.196	2.061	0.196	2.061
0	0	20	50	0.288	4.168	0.196	2.061	0.196	2.061	0.196	2.061
0	0	50	20	0.255	2.260	0.196	2.061	0.196	2.061	0.196	2.061
0	0	50	50	0.328	4.136	0.196	2.061	0.196	2.061	0.196	2.061
20	20	20	20	0.220	2.469	0.195	2.233	0.125	2.132	0.288	2.207
20	20	20	50	0.287	4.523	0.195	2.233	0.125	2.132	0.288	2.207
20	20	50	20	0.255	2.451	0.195	2.233	0.125	2.132	0.288	2.207
20	20	50	50	0.328	4.492	0.195	2.233	0.125	2.132	0.288	2.207
20	50	20	20	0.262	2.983	0.227	2.707	0.109	2.393	0.370	3.007
20	50	20	50	0.354	5.426	0.227	2.707	0.109	2.393	0.370	3.007
20	50	50	20	0.303	2.962	0.227	2.707	0.109	2.393	0.370	3.007
20	50	50	50	0.401	5.390	0.227	2.707	0.109	2.393	0.370	3.007
50	20	20	20	0.259	2.986	0.224	2.709	0.121	2.354	0.337	2.678
50	20	20	50	0.347	5.441	0.224	2.709	0.121	2.354	0.337	2.678
50	20	50	20	0.299	2.964	0.224	2.709	0.121	2.354	0.337	2.678
50	20	50	50	0.395	5.401	0.224	2.709	0.121	2.354	0.337	2.678
50	50	20	20	0.243	3.703	0.209	3.349	0.038	2.795	0.362	3.401
50	50	20	50	0.332	6.744	0.209	3.349	0.038	2.795	0.362	3.401
50	50	50	20	0.286	3.682	0.209	3.349	0.038	2.795	0.362	3.401
50	50	50	50	0.383	6.703	0.209	3.349	0.038	2.795	0.362	3.401
True value				0.25	2.0	0.25	2.0	0.25	2.0	0.25	2.0

*GSD* geometric standard deviation, *ERR* excess relative risk.

## Discussion

We have demonstrated that the coverage probabilities of all methods for the linear coefficient $\alpha$ are near the desired 95% level, irrespective of the magnitudes of assumed Berkson and classical error, whether shared or unshared (Table 2). The coverage probabilities for the quadratic coefficient $\beta$ are generally too low for the unadjusted and regression calibration methods, particularly for larger magnitudes of Berkson error (with GSD = 50%), whether this is shared or unshared; by contrast the coverage probabilities for $\beta$ using MCML are uniformly too high (Table 2). The extended regression calibration method yields generally more satisfactory coverage probabilities, in most cases better than the other methods (Table 2). The reason for the coverage probabilities of the quadratic coefficient $\beta$ being unsatisfactory may be related to the fact that for all methods apart from extended regression calibration the estimates of this parameter are upwardly biased, much more substantially so than for $\alpha$ (Table 3). The fact that $\beta$ may not be well estimated implies that assessments of curvature may be incorrect, and in particular may result in overestimation of the degree of curvature in the dose response, at least for the scenarios investigated here.

An unexpected feature of our analysis is that when there is only classical error the unadjusted analysis (using surrogate dose) can result in appreciable upward bias, contrary to what is often seen when there is pure classical error (Table 3). In this case the ratio of doses (surrogate to true) is approximately lognormal (Fig. 1) and for each simulation the ratio is generally much the same in all dose groups except the topmost one, suggesting that it is the shared classical error that is dominating—the unshared error averages out in general, although it does contribute somewhat to the topmost group (data not shown). Although the distribution of fitted $\hat{\alpha }$ and $\hat{\beta }$ to some extent reflect this, as shown in Fig. 2 the distributions of both optimal $\hat{\alpha }$ and $\hat{\beta }$ are markedly skew, with pronounced upper tail, particularly for $\hat{\beta }$. This results in pronounced upward bias in the mean estimates of $\hat{\alpha }$ and $\hat{\beta }$ for the unadjusted (surrogate dose) analysis (Fig. 2). The reason for the skewness of the fitted $\hat{\alpha }$ and $\hat{\beta }$ is reasonably obvious—given the range of true doses generated (up to the level of about 2 Gy), the $\hat{\alpha }$ and $\hat{\beta }$ cannot be very substantially negative without the relative risk for the higher dose groups becoming negative, which would lead to the likelihood blowing up. It should also be noted that when there is only classical error, as implied by expression (1) all true doses used for regression calibration, extended regression calibration and MCML are precisely the central estimates given in Table 1. This implies that in this case regression calibration and MCML will yield precisely the same regression coefficients. Since the covariance term that is used to adjust the likelihood for extended regression calibration becomes trivial (i.e., 0), the second order likelihood adjustment term in Appendix A expression (A3) drops out, and extended regression calibration reduces to the standard type of calibration.

The defects in regression calibration that our modelling has revealed are not too surprising, as it is well known that this method can break down when dose error is substantial^33^, as it is in many of our scenarios. The essence of regression calibration is to replace of the vector of true doses $(D_{i} )$ in the expression for the theoretical likelihood $L[(y_{i} ),\vartheta ,(D_{i} ),(Z_{i} )]$ by the vector of conditional expectations $(E[D_{i} |d_{i} ,Z_{i} ))$ of true dose $(D_{i} )$ given the nominal or observed dose $(d_{i} )$ and ancillary variables $(Z_{i} )$. The method is relatively simple to apply, although it does require some method of determining the magnitude of dose error, as well as the distribution of true dose in the data. However, the distribution of true dose can be determined to some extent via deconvolution of the distribution of nominal dose. The method has the considerable advantage that once the conditional expectations have been derived conventional statistical software can be used to perform regressions. The method has been successfully applied to the LSS cohort by a number of investigators^16–20,42^ and has also been used in a few other radiation exposed groups^26^. There have also been extensive applications in the non-radiation literature, reviewed by Carroll et al.^33^ and more recently in a series of papers by Shaw et al.^38,39^. Calibration approaches that take account of mixtures of Berkson and classical error have also been developed and used to fit domestic radon case–control data^21^.

The relatively poor performance of MCML is perhaps more surprising. MCML relies on replacing the likelihood, as a function of the true dose vectors $(D_{i} )$, by its expectation with respect to the nominal dose array $(d_{i} )$, $E\left[ {L[(y_{i} ),\vartheta ,(D_{i} ),(d_{i} )]|(d_{i} )} \right] = \int {L[(y_{i} ),\vartheta ,(D_{i} ),(d_{i} )]} \;dP(D_{i} |d_{i} )$. The marginal likelihood thus derived can then be used for likelihood-based inference in the usual way^43^. The integration is often achieved via Monte Carlo samples, produced from a Monte Carlo dosimetry system (MCDS) that can simulate true doses based on often quite complex dosimetric models, which can incorporate uncertainties in many dosimetric and other parameters. Implementation of MCML relies on specialist software, often written in high level languages such as Fortran or C/C++, and is generally highly computationally burdensome. It may suffer from the additional problem occasioned by attempting to sample from very high dimensional distributions, the so-called curse of dimensionality, which implies that a large part of the overall distribution of true dose will not have been sampled. However, whether this is a problem in practice is not always altogether clear—for example the underlying set of parameters being sampled may be in some cases quite low dimensional. In particular, the Monte Carlo simulations inspired by the Mayak worker data exhibit little evidence of upward bias, at most 15% or so, arguably of little material significance given the uncertainties^44^. Even where such problems may arise there may be ways round this, for example by using importance Monte Carlo sampling, as outlined by Dai et al.^45^. MCML has been used for analysis of nuclear workers^46^, indoor radon data^47^ and in a number of studies of Chernobyl-exposed groups^25–27,31^, and in a few other datasets^48^. The poor performance of MCML in our study may reflect the fact that there is hidden correlation within each group, which MCML cannot take into account, given the collapsed nature of the data that we use.

A Bayesian approach to the measurement error problem has been developed over the last 30 years which rests on the formulation of conditional independence relationships between different model components^49,50^, following the general structure outlined by Clayton^51^. In this approach three basic sub-models are distinguished and linked: the disease model, the measurement model and the exposure model. The power of this Bayesian approach, as with MCML, is that the dosimetric uncertainty is (in principle) reflected in the variability of the model parameters relating dose to health effects. An adapted Bayesian method of correction for measurement error has been fitted to various versions of the LSS mortality data^22–24,30^, also to an older version of the LSS incidence data^23^. Derivation of the posterior distribution is generally analytically infeasible, and relies on the MCMC algorithm, specifically the Metropolis sampler, which converges to the posterior distribution of the risk parameters. However, the speed of convergence is not known, and in practice one relies on a number of ad hoc tests of convergence such as the Brooks-Gelman-Rubin statistic^52,53^ and other less formal methods, e.g., inspection of caterpillar plots. As such all one can know is when convergence has not taken place. Flexible and efficient software exists to run this on a number of platforms e.g., OpenBUGS^54^ or rjags^55^. The method is exceptionally computationally burdensome. As with all Bayesian methods the choice of prior is critical.

Some other methods of more limited utility have been developed for dealing with dosimetric error, which we briefly review. The simulation-extrapolation (SIMEX) method was developed by Cook and Stefanski^56^. It was originally proposed for datasets where the error is of pure classical form, and where the precise magnitude of the dose error is known. The method proceeds by adding classical random error with progressively larger GSD to the nominal dose estimates, performing regression analyses, this Monte Carlo procedure being repeated a large number of times to reduce sampling uncertainties. A curve is then fitted to the regression estimates as a function of magnitude of dose error, and the curve used to extrapolate back to 0 error. It is computationally highly intensive. R packages exist (e.g. **simex**^57^) to fit at least certain types of generalised linear model^41^ although not the linear relative risk models in common use in epidemiological analysis of radio-epidemiological data. Quite apart from the computational difficulties, the method relies on a substantial extrapolation (from the given level of dose error to 0 error), a jump that may be difficult to justify. An attempt has been made to expand SIMEX to allow for a mixture of classical and Berkson errors utilising the LSS data^37^. Perhaps due to the computational cost with the cross-tabulation and because of the limited types of error structure that can be handled it has been used only twice to our knowledge, in analysis of the LSS data^28,37^.

The so-called two dimensional Monte Carlo using Bayesian model averaging (2DMC-BMA) method relies on Monte Carlo simulations from an MCDS. The key aspect is that ensembles of doses $(D_{ijk} )_{j = 1\,\;k = 1}^{{N\;\;n_{j} }}$ are produced for all individuals for a large number of scenarios $i$, $1 \le i \le M$. However, unlike other uses of MCDS it is assumed that only one of the dose scenarios $i$, and therefore one of the sets of dose realisations $(D_{ijk} )_{j = 1\,\;k = 1}^{{N\;\;n_{j} }}$ is the correct one. Essentially this method therefore assumes something like a combination of functional and structural approaches—there are assumed to be random errors in the data, but certain parameters are assumed fixed (but unknown). The BMA approach is used to reweight the scenarios depending on the goodness of fit^29^. So realisations where the risk-dose relationship was linear would be much more highly weighted than realisations where this was not the case. The contrast with MCML is quite pronounced—MCML works by averaging the likelihood in one go and then maximising the averaged likelihood with respect to the parameters of interest. The 2DMC-BMA method appears designed for applications where there is a substantial amount of shared error. This method has been applied to analysis of thyroid nodules in a dataset of persons exposed to atmospheric weapons tests in the former Soviet Union^58^. The method has been much discussed^44^. Stram et al.^44^ suggested that the method will produce substantially upwardly biased estimates of risk, also that the coverage may be poor. The implementation of the methodology presently relies on proprietary software, and has only been used by the group that developed it. Another substantial problem with the method is the use of BMA, reflecting general criticism made of this class of models in the literature^59,60^. An implicit assumption of BMA is that one of the underlying models is the “true” one with convergence guaranteed to the “true” model^61^. As with all Bayesian methods the choice of prior is critical.

Zhang et al.^62^ developed their corrected information matrix (CIM) method for analysis of datasets where there is pure Berkson error in radiation dose, a substantial part of it shared. This entails an extensive calculation, which requires specially written software, which the authors have developed in Python^63^ specifically applied to the Mayak worker lung cancer data. R code has also been developed for fitting this model to US radiologic technologists (USRT) cataract data for relative risk and absolute risk Poisson models^64^. The calculations result in inflation of the confidence intervals (CI) on the regression estimate—the central estimate is largely unchanged. Arguably the assumptions underlying the CIM method, that all dose simulations are samples from the true dose, may be unlikely, but this assumption is arguably less implausible than that made for 2DMC-BMA, which assumes that one realisation is true. The method appears to be well adapted to analysis of the Mayak data^63^, where there is a substantial amount of shared error. In the USRT cataract data, the amount of shared error is small, and the method yields largely trivial adjustments to CI^64^.

A relatively novel method of measurement error correction has been recently introduced, moment reconstruction (MR)^65^. The basic idea is that one substitutes for the nominal dose estimate $d_{i}$ a new quantity $M_{{d_{i} ,Y_{i} }}$ which is chosen to have the same first two moments (with the outcome variable $Y_{i}$) of the joint distribution as $(D_{i} ,Y_{i} )$. It can be shown^65^ that the solution is given by $M_{{d_{i} ,Y_{i} }} = E[d_{i} |Y_{i} ](1 - G) + d_{i} G$ where $G = G(Y) = {\text{cov}} [D_{i} |Y_{i} ]^{0.5} ({\text{cov}} [d_{i} |Y_{i} ])^{ - 0.5}$. Under linear regression it is easily shown that MR is entirely equivalent to regression calibration^65^. It has the advantage over regression calibration that it yields consistent estimates even when the model is non-linear, or when the errors in dose are non-differential^65^. Moment-adjusted imputation (MAI) is a generalisation of MR, in which the moments of $(D_{i} ,Y_{i} )$ are matched by $M_{{d_{i} ,Y_{i} }}$, usually up to at least the 4th order^66,67^. However, both MR and MAI require knowledge of second and higher order moments of the true dose distribution in conjunction with the disease endpoint, information that would generally have to come from a gold standard sample, which is not often available in radiation studies. Although MR and MAI can be more efficient than regression calibration there are circumstances when efficiency is reduced compared with regression calibration^39^. Perhaps for all these reasons, to the best of our knowledge neither method has been used in radiation applications.

## Conclusions

We have outlined a modification of the regression calibration method^33^ which is particularly suited to studies where there is a substantial amount of shared error, and where there may also be curvature in the true dose response. We have shown in fits to a number of synthetic datasets in which there is substantial upward curvature in the true dose response, and varying (and sometimes substantial) amounts of classical and Berkson error, that the coverage probabilities of all methods for the linear coefficient are near the desired level, irrespective of the magnitudes of assumed Berkson and classical error, whether shared or unshared. However, the coverage probabilities for the quadratic coefficient are generally too low for the unadjusted and regression calibration methods, particularly for larger magnitudes of the Berkson error, whether this is shared or unshared, while MCML yields coverage probabilities for the quadratic coefficient that are uniformly too high. The extended regression calibration method yields coverage probabilities that are too low when shared and unshared Berkson errors are both large, although otherwise it performs well, and coverage is generally better than these other methods. A notable feature is that for all methods apart from extended regression calibration the estimates of the quadratic coefficient are substantially upwardly biased.

### Supplementary Information

Supplementary Information 1.

Supplementary Information 2.

## Data Availability

The datasets generated and analysed in the current study are available by running the Fortran 95/2003 program fitter_shared_error_simulation_reg_cal.for, given in the online web repository, with any of the five steering input files given there. All are described in Appendix B. The datasets are temporarily stored in computer memory, and the program uses them for fitting the Poisson models described in the “Methods” section.
