# Supplementary material for: A generalisation of the method of regression calibration
Source: Sci Rep. 2023 Sep 13;13:15127. doi: 10.1038/s41598-023-42283-y (PMC10499875; doi:10.1038/s41598-023-42283-y)
Supplement: Supplementary file 1 — Supplementary Information 1. [file 41598_2023_42283_MOESM1_ESM.docx]

## **Appendix A. Statistical considerations relating to regression calibration and Monte Carlo maximum likelihood**

When one has a group of individuals with outcomes $(y_{i})$ (e.g. cancer) that are a function of unknown true doses $(D_{i})$ the overall likelihood is given by $L[(y_{i}),\vartheta,(D_{i})]$, where $\vartheta$ is some vector of unknown parameters, which will include (but in general not be limited to) regression coefficients in dose. [For notational simplicity we omit vectors of ancillary variables $(Z_{i})$ from the likelihood.] The likelihood, being a function of unknown quantities, cannot in general be evaluated. However, if we have nominal observed doses $(d_{i})$ then we can evaluate the marginal likelihood:

$E\left[L[(y_{i}),\vartheta,(D_{i}),(d_{i})]|(d_{i})\right]=\int L[(y_{i}),\vartheta,(D_{i}),(d_{i})] dP(D_{i}|d_{i})$ (A1)

In particular this expression forms the basis of Monte Carlo maximum likelihood, when the integral on the right hand side is replaced by a sum over Monte Carlo realisations $(D_{i})|(d_{i})$. $\vartheta$ can then be maximised in the usual way, and the usual likelihood machinery applied to perform inference ^1^. By Taylor’s theorem we may expand the likelihood function $L[(y_{i}),\vartheta,(D_{i})]$:

$\begin{matrix} L[(y_{i}),\vartheta,(D_{i}),(d_{i})]=L[(y_{i}),\vartheta,E[(D_{i})|(d_{i})],(d_{i})]+\sum_{i} (D_{i}-E[D_{i}|(d_{k})])\left.\frac{\partial L[(y_{i}),\vartheta,(D_{i}),(d_{i})]}{\partial D_{i}}\right|_{E[D_{i}|(d_{k})]} \\ +(1/2)\sum_{i,j} (D_{i}-E[D_{i}|(d_{k})])(D_{j}-E[D_{j}|(d_{k})])\left.\frac{\partial^{2}L[(y_{i}),\vartheta,(D_{i}),(d_{i})]}{\partial D_{i}\partial D_{j}}\right|_{E[D_{i}|(d_{i})]}+O((D_{i}-E[D_{i}|(d_{k})])^{3}) \end{matrix}$ (A2)

Therefore the marginal likelihood, analogous to (A1), obtained by taking conditional expectations of both sides in (A2) can be written:

$\begin{matrix} E\left[\left.L[(y_{i}),\vartheta,(D_{i}),(d_{i})]\right|(d_{k})\right]=L[(y_{i}),\vartheta,E[(D_{i})|(d_{i})],(d_{k})] \\ +\sum_{i} E\left[\left.(D_{i}-E[D_{i}|(d_{k})]\left.\frac{\partial L[(y_{i}),\vartheta,(D_{i}),(d_{i})]}{\partial D_{i}}\right|_{E[D_{i}|(d_{k})]}\right|(d_{k})\right] \\ +(1/2)\sum_{i,j} E\left[\left.(D_{i}-E[D_{i}|(d_{k})])(D_{j}-E[D_{j}|(d_{k})])\left.\frac{\partial^{2}L[(y_{i}),\vartheta,(D_{i}),(d_{i})]}{\partial D_{i}\partial D_{j}}\right|_{E[D_{i}|(d_{i})]}\right|(d_{k})\right] \\ +O(E[(D_{i}-E[D_{i}|(d_{k})])^{3}]) \\ =L[(y_{i}),\vartheta,E[(D_{i})|(d_{i})],(d_{i})] \\ +(1/2)\sum_{i,j} E[(D_{i}-E[D_{i}|(d_{k})])(D_{j}-E[D_{j}|(d_{k})])|(d_{k})]\left.\frac{\partial^{2}L[(y_{i}),\vartheta,(D_{i}),(d_{i})]}{\partial D_{i}\partial D_{j}}\right|_{E[D_{i}|(d_{i})]} \\ +O(E[(D_{i}-E[D_{i}|(d_{k})])^{3}]) \end{matrix}$(A3)

This makes use of the fact that:

$\begin{matrix} E\left[\left.(D_{i}-E[D_{i}|(d_{k})]\left.\frac{\partial L[(y_{i}),\vartheta,(D_{i}),(d_{i})]}{\partial D_{i}}\right|_{E[D_{i}|(d_{k})]}\right|(d_{k})\right] \\ \left.=\frac{\partial L[(y_{i}),\vartheta,(D_{i}),(d_{i})]}{\partial D_{i}}\right|_{E[D_{i}|(d_{k})]}E\left[\left.(D_{i}-E[D_{i}|(d_{k})]\right|(d_{k})\right]=0 \end{matrix}$ (A4)

If we can neglect the quadratic and higher order terms in $D_{i}-E[D_{i}|(d_{k})]$ the marginal likelihood (A3) therefore reduces to:

$L[(y_{i}),\vartheta,E[(D_{i})|(d_{i})],(d_{i})]$ (A5)

In this expression we therefore simply substitute true dose $(D_{i})$in the likelihood by the conditional expectation of the true dose with respect to the nominal dose $(d_{i})$, $E[(D_{i})|(d_{i})]$. This is the basis of regression calibration ^2^. If either the dose errors or the regression coefficient in dose are large then the quadratic terms cannot be neglected. In particular these quadratic terms may include non-trivial terms for the interindividual correlations of the $(D_{i})$.

When the quadratic terms in dose cannot be neglected we can use (A3) to derive a correction to the plug in term for the marginal likelihood $L[(y_{i}),\vartheta,E[(D_{i})|(d_{i})],(d_{i})]$ to make it more nearly approximate the true marginal likelihood $E\left[\left.L[(y_{i}),\vartheta,(D_{i}),(d_{i})]\right|(d_{k})\right]$.

It should be noted that the error terms in (A3) can be to some extent numerically evaluated. For example, suppose that a Poisson model is assumed for disease cases or deaths, so that for individual $i$ the expected number of cases/deaths is given by a linear-quadratic relative risk model:

$e_{i}=PY_{i}f(Z_{i},(\theta_{i})_{2}^{n})[1+\theta_{0}D_{i}+\theta_{1}D_{i}^{2}]$ (A6)

## where $PY_{i}$ is the number of person years, the baseline disease rate is given by a specified parametric function $f()$ of a vector of covariates $(Z_{i})$and a vector of unknown parameters $(\theta_{i})_{0}^{n}$. Assuming that $n_{i}$ is the number of deaths/cases then we may write the likelihood as:

$L[(y_{i}),\theta,(D_{i}),(Z_{i})]=\prod_{i=1}^{N} \frac{e_{i}^{n_{i}}\exp[-e_{i}]}{n_{i}!}$ (A7)

Then:

$\frac{\partial L[(y_{i}),\theta,(D_{i}),(Z_{i})]}{\partial D_{k}}=\sum_{i=1}^{N} (n_{i}/e_{i}-1)\frac{\partial e_{i}}{\partial D_{k}}\prod_{j=1}^{N} \frac{e_{j}^{n_{j}}\exp[-e_{j}]}{n_{j}!}$ (A8)

and:

$\begin{matrix} \frac{\partial^{2}L[(y_{i}),\theta,(D_{i}),(Z_{i})]}{\partial D_{k}\partial D_{l}}=\left[\prod_{m=1}^{N} \frac{e_{j}^{n_{m}}\exp[-e_{m}]}{n!}\right]\text{x} \\ \left(\sum_{i=1}^{N} -n_{i}/e_{i}^{2}\frac{\partial e_{i}}{\partial D_{l}}\frac{\partial e_{i}}{\partial D_{k}}+\sum_{i=1}^{N} (n_{i}/e_{i}-1)\frac{\partial^{2}e_{i}}{\partial D_{k}\partial D_{l}}+\sum_{i,j=1}^{N} (n_{i}/e_{i}-1)(n_{j}/e_{j}-1)\frac{\partial e_{i}}{\partial D_{k}}\frac{\partial e_{j}}{\partial D_{l}}\right) \end{matrix}$ (A9)

We can straightforwardly evaluate many parts of this expression. From (A6) we have:

$\frac{\partial e_{i}}{\partial D_{j}}=1_{i=j}PY_{i}f(Z_{i},(\theta_{i})_{2}^{n})[\theta_{0}+2\theta_{1}D_{i}]$ (A10)

$\frac{\partial^{2}e_{i}}{\partial D_{j}\partial D_{k}}=1_{i=j=k}2PY_{i}f(Z_{i},(\theta_{i})_{2}^{n})\theta_{1}$ (A11)

since only the dose to each individual cell is assumed (by (A6)) to affect that individual cell. This implies that (A8) and (A9) simplify considerably, as follows:

$\begin{matrix} \frac{\partial L[(y_{i}),\theta,(D_{i}),(Z_{i})]}{\partial D_{k}}=(n_{k}/e_{k}-1)\frac{\partial e_{k}}{\partial D_{k}}\prod_{j=1}^{N} \frac{e_{j}^{n_{j}}\exp[-e_{j}]}{n_{j}!} \\ =(n_{k}/e_{k}-1)PY_{k}f(Z_{k},(\theta_{i})_{2}^{n})[\theta_{0}+2\theta_{1}D_{k}]\prod_{j=1}^{N} \frac{e_{j}^{n_{j}}\exp[-e_{j}]}{n_{j}!} \end{matrix}$ (A12)

and:

$\begin{matrix} \frac{\partial^{2}L[(y_{i}),\theta,(D_{i}),(Z_{i})]}{\partial D_{k}\partial D_{l}}=\left[\prod_{m=1}^{N} \frac{e_{j}^{n_{m}}\exp[-e_{m}]}{n!}\right]\text{ x } \\ \left(-n_{k}/e_{k}^{2}\left[\frac{\partial e_{k}}{\partial D_{k}}\right]^{2}1_{k=l}+(n_{k}/e_{k}-1)\frac{\partial^{2}e_{k}}{\partial D_{k}^{2}}1_{k=l}+(n_{k}/e_{k}-1)(n_{l}/e_{l}-1)\frac{\partial e_{k}}{\partial D_{k}}\frac{\partial e_{l}}{\partial D_{l}}\right) \\ =\left[\prod_{m=1}^{N} \frac{e_{j}^{n_{m}}\exp[-e_{m}]}{n!}\right]\text{ x } \\ \left(\begin{matrix} -n_{k}/e_{k}^{2}\left[PY_{k}f(Z_{k},(\theta_{i})_{2}^{n})[\theta_{0}+2\theta_{1}D_{k}]\right]^{2}1_{k=l}+(n_{k}/e_{k}-1)2PY_{k}f(Z_{k},(\theta_{i})_{2}^{n})\theta_{1}1_{k=l} \\ +(n_{k}/e_{k}-1)(n_{l}/e_{l}-1)\left[PY_{k}f(Z_{k},(\theta_{i})_{2}^{n})[\theta_{0}+2\theta_{1}D_{k}]\right]\left[PY_{l}f(Z_{l},(\theta_{i})_{2}^{n})[\theta_{0}+2\theta_{1}D_{l}]\right] \end{matrix}\right) \end{matrix}$(A13)

All expressions in (A12) or (A13) are then evaluated at $D_{i}=E[D_{i}|(d_{k})]$ and $D_{j}=E[D_{j}|(d_{k})]$for the purposes of plugging into expression (A3). The covariance of the $D_{i}-E[D_{i}|(d_{k})]$ and $D_{j}-E[D_{j}|(d_{k})]$ can be straightforwardly derived from the output of a Monte Carlo dosimetry system, if such a thing exists. If the error distributions are known by other means (e.g. analytical or theoretical understanding of the dosimetry) the necessary low dimensional (2D) numerical integrals can be evaluated, possibly numerically, e.g., using Bulirsch-Stoer or other adaptive numerical integrators ^3^.

**References**

1 Schervish, M. J. *Theory of statistics*. (Springer Verlag, 1995).

2 Carroll, R. J., Ruppert, D., Stefanski, L. A. & Crainiceanu, C. M. 1-488 (Chapman and Hall/CRC, Boca Raton, FL, 2006).

3 Press, W. H., Teukolsky, S. A., Vetterling, W. T. & Flannery, B. P. 1-934 (Cambridge University Press, Cambridge, 1992).
