# Supplementary material for: A generalisation of the method of regression calibration
Source: Sci Rep. 2023 Sep 13;13:15127. doi: 10.1038/s41598-023-42283-y (PMC10499875; doi:10.1038/s41598-023-42283-y)
Supplement: Supplementary file 2 — Supplementary Information 2. [file 41598_2023_42283_MOESM2_ESM.zip › Appendix B header description.docx]

**Appendix B. Fortran 95-2003 program used to generate simulated doses and perform model fitting, and steering input file**

The datasets generated and analysed in the current study are available by running the Fortran 95/2003 program **fitter_shared_error_simulation_reg_cal.for**, given in the online web repository, with any of the five steering input files given there:
fit_shared_error_simulation_reg_cal_unshared_Berkson_0%_shared_Berkson_0%_lin_quad.inp

fit_shared_error_simulation_reg_cal_unshared_Berkson_20%_shared_Berkson_20%_lin_quad.inp

fit_shared_error_simulation_reg_cal_unshared_Berkson_20%_shared_Berkson_50%_lin_quad.inp

fit_shared_error_simulation_reg_cal_unshared_Berkson_50%_shared_Berkson_20%_lin_quad.inp

fit_shared_error_simulation_reg_cal_unshared_Berkson_50%_shared_Berkson_50%_lin_quad.inp

The datasets are temporarily stored in computer memory, and the program uses them for fitting the Poisson models described in the Methods section.
